# Mechanism of H_2_S Oxidation by the Dissimilatory Perchlorate-Reducing Microorganism *Azospira suillum* PS

**DOI:** 10.1128/mBio.02023-16

**Published:** 2017-02-21

**Authors:** Misha G. Mehta-Kolte, Dana Loutey, Ouwei Wang, Matthew D. Youngblut, Christopher G. Hubbard, Kelly M. Wetmore, Mark E. Conrad, John D. Coates

**Affiliations:** a Energy Biosciences Institute, University of California Berkeley, Berkeley, California, USA; b Plant and Microbial Biology Department, University of California Berkeley, Berkeley, California, USA; c Earth and Environmental Sciences Area, Lawrence Berkeley National Laboratory, Berkeley, CA, USA; Oregon State University

## Abstract

The genetic and biochemical basis of perchlorate-dependent H_2_S oxidation (PSOX) was investigated in the dissimilatory perchlorate-reducing microorganism (DPRM) *Azospira suillum* PS (PS). Previously, it was shown that all known DPRMs innately oxidize H_2_S, producing elemental sulfur (S^o^). Although the process involving PSOX is thermodynamically favorable (*ΔG*°′ = −206 kJ ⋅ mol^−1^ H_2_S), the underlying biochemical and genetic mechanisms are currently unknown. Interestingly, H_2_S is preferentially utilized over physiological electron donors such as lactate or acetate although no growth benefit is obtained from the metabolism. Here, we determined that PSOX is due to a combination of enzymatic and abiotic interactions involving reactive intermediates of perchlorate respiration. Using various approaches, including barcode analysis by sequencing (Bar-seq), transcriptome sequencing (RNA-seq), and proteomics, along with targeted mutagenesis and biochemical characterization, we identified all facets of PSOX in PS. In support of our proposed model, deletion of identified upregulated PS genes traditionally known to be involved in sulfur redox cycling (e.g., Sox, sulfide:quinone reductase [SQR]) showed no defect in PSOX activity. Proteomic analysis revealed differential abundances of a variety of stress response metal efflux pumps and divalent heavy-metal transporter proteins, suggesting a general toxicity response. Furthermore, *in vitro* biochemical studies demonstrated direct PSOX mediated by purified perchlorate reductase (PcrAB) in the absence of other electron transfer proteins. The results of these studies support a model in which H_2_S oxidation is mediated by electron transport chain short-circuiting in the periplasmic space where the PcrAB directly oxidizes H_2_S to S^o^. The biogenically formed reactive intermediates (ClO_2_^−^ and O_2_) subsequently react with additional H_2_S, producing polysulfide and S^o^ as end products.

## IMPORTANCE

Inorganic sulfur compounds are widespread in nature, and microorganisms are central to their transformation, thereby playing a key role in the global sulfur cycle. Sulfur oxidation is mediated by a broad phylogenetic diversity of microorganisms, including anoxygenic phototrophs and either aerobic or anaerobic chemotrophs coupled to oxygen or nitrate respiration, respectively. Recently, perchlorate-respiring microorganisms were demonstrated to be innately capable of sulfur oxidation regardless of their phylogenetic affiliation. As recognition of the prevalence of these organisms intensifies, their role in global geochemical cycles is being queried. This is further highlighted by the recently recognized environmental pervasiveness of perchlorate not only across Earth but also throughout our solar system. The inferred importance of this metabolism not only is that it is a novel and previously unrecognized component of the global sulfur redox cycle but also is because of the recently demonstrated applicability of perchlorate respiration in the control of biogenic sulfide production in engineered environments such as oil reservoirs and wastewater treatment facilities, where excess H_2_S represents a significant environmental, process, and health risk, with associated costs approximating $90 billion annually.

## INTRODUCTION

Inorganic sulfur compounds are widespread in nature, and microorganisms are central to their transformation, thereby playing a key role in the global sulfur cycle (1). Given the complexity of the sulfur compounds, it is not always easy to separate microbially mediated reactions from purely abiotic reactions. Microbial transformation of sulfur can be broadly classified as either direct or indirect. Microorganisms contribute to the direct sulfur cycle either by assimilation for biosynthesis of cell constituents or by dissimilation for energy generation (2). As an electron donor for dissimilation, environmental H_2_S is oxidized to sulfate by sulfur-oxidizing bacteria (3–5). Alternatively, H_2_S is transformed through abiotic interaction with metals such as Fe(III) and Mn(IV) (6). Microbial sulfur oxidation pathways have been studied in a variety of phylogenetically diverse microorganisms using a plethora of biochemical and molecular methods (2). This metabolism is mediated by anoxygenic phototrophs and either aerobic or anaerobic chemotrophs coupled to oxygen or nitrate respiration, respectively (7). Recently, anaerobic H_2_S oxidation coupled to perchlorate reduction was identified as an innate capacity of dissimilatory perchlorate-reducing microorganisms (DPRMs) (8, 9). As recognition of the prevalence of microbial perchlorate respiration intensifies, the role of DPRM in global geochemical cycles is being queried (10). This is emphasized by the recently inferred environmental pervasiveness of perchlorate not only across Earth but also throughout our solar system and possibly beyond (10, 11). Apart from their obvious and primary role in the diagenesis of chlorine oxyanions, DPRMs have also been shown to be metabolically versatile, with the capacity to utilize a diversity of both organic and inorganic electron donors (12). Unlike the majority of known sulfur-oxidizing chemotrophs, H_2_S oxidation by DPRMs is incomplete, producing intra- and extracellular elemental sulfur globules as the primary end products of the metabolism (8, 9). While growth is not associated with the metabolism, H_2_S is preferentially utilized over physiological organic substrates (9), suggesting that H_2_S oxidation by DPRMs may predominantly be a detoxification strategy. Although the environmental prevalence of this metabolism is currently unknown, DPRMs appear to be ubiquitous in soil, sediment, and aqueous environments (12). The inferred importance of this metabolism is not only that it is a novel and previously unrecognized component of the global sulfur redox cycle but also is because of the recent demonstrated applicability of perchlorate and DPRMs in the control of biogenic sulfide production in engineered environments such as oil and gas reservoirs and wastewater treatment facilities, where excess H_2_S represents a significant environmental, process, and health risk (9, 10, 13). While many DPRMs can alternatively utilize nitrate for heterotrophic metabolism, H_2_S oxidation is a perchlorate-dependent form of metabolism in these organisms, suggesting that it involves some unique components of the perchlorate respiratory pathway (8, 9). This perchlorate-specific H_2_S oxidation could be due to a combination of enzymatic and abiotic interactions with the reactive chlorine species (RCS) or molecular oxygen generated as intermediates of canonical perchlorate respiration (12). These abiotic reactions may have integrated with enzymatic steps as a part of a metabolic strategy or may solely be an inadvertent consequence of generating highly oxidized intermediates in a highly reduced anoxic environment. An analogous hybrid enzymatic-abiotic model was previously identified for nitrate-dependent Fe(II) oxidation (14, 15). Although supportive of the phenotypic, morphological, and analytical data available on H_2_S oxidation by DPRMs (9), the underlying genetic and biochemical mechanisms require elucidation.

The current study explored PSOX in the model DPRM *Azospira suillum* PS (PS) using a systems biology approach incorporating phenotypic characterization, molecular biology, random barcode TN-seq (RB TN-seq) analysis, transcriptomic and proteomic profiling, and biochemical characterization. Strain PS is a facultative anaerobe capable of respiring nitrate or perchlorate under anoxic conditions and has a tractable genetic system (16).

## RESULTS

### Perchlorate reduction is essential for sulfide oxidation by PS.

**FIG 1:**
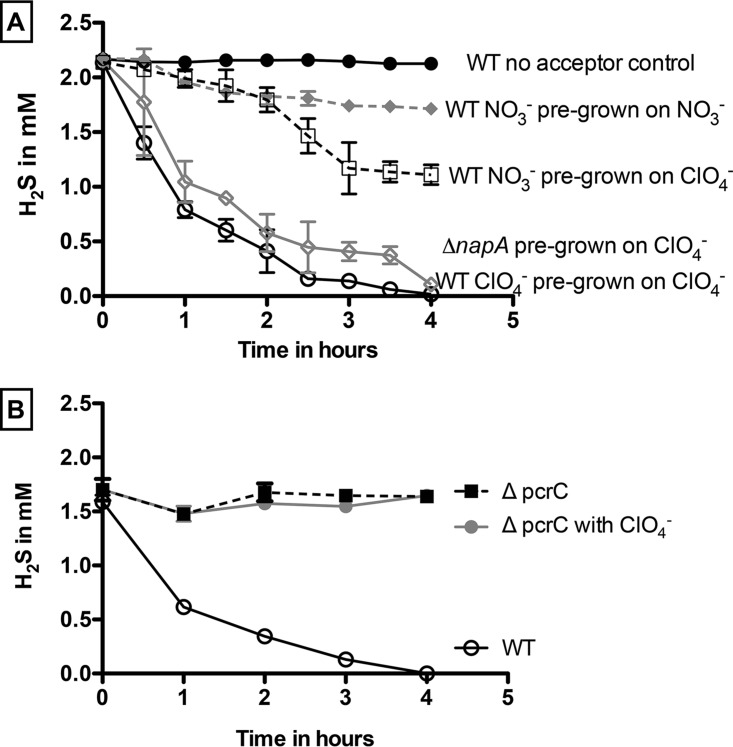
Sulfide oxidation coupled to perchlorate or nitrate reduction by washed cell suspensions of wild-type *Azospira suillum* pregrown on perchlorate or nitrate and Δ*napA* mutants pregrown on perchlorate (A) and by the wild-type strain and *ΔpcrA* mutants pregrown on nitrate (B).

Washed cell suspensions of wild-type (WT) PS completely oxidized 2.15 mM H_2_S (0.47 ± 0.04 mM H_2_S h^−1^) within 5 h in the absence of any additional organic electron donor when pregrown on perchlorate (10 mM) (Fig. 1A and Fig. S1 in the supplemental material). As before (9), no H_2_S was oxidized in heat-killed controls or in controls lacking perchlorate. Some oxidation (~0.75 mM) was observed in perchlorate-grown cells at a lower rate (0.31 ± 0.02 mM H_2_S h^−1^) when nitrate was provided as the electron acceptor (Fig. 1A). In this case, H_2_S oxidation did not go to completion despite an excess of nitrate. This nitrate-dependent oxidation was presumably due to nitrate turnover mediated by the perchlorate reduction pathway present in these cells. Such nitrate turnover mediated by the perchlorate reduction pathway was previously observed for the DPRM *Dechloromonas agitata* strain CKB, which does not contain a known pathway for nitrate respiration and is incapable of growth coupled to nitrate reduction (12, 17). Furthermore, our recent biochemical studies on PS demonstrated that purified perchlorate reductase can alternatively reduce nitrate to nitrite (18). In support of this conclusion, minimal and transient H_2_S oxidation (0.12 ± 0.01 mM H_2_S h^−1^) occurred when washed cell suspensions pregrown on nitrate to prevent expression of the perchlorate reduction pathway were incubated with H_2_S and nitrate (Fig. 1A). To further confirm that H_2_S oxidation is specific to perchlorate respiration, a deletion of the nitrate reductase NapA subunit in PS (Δ*napA*) was constructed. Although the Δ*napA* mutant was incapable of growth on nitrate, a washed cell suspension pregrown on perchlorate showed no defect in H_2_S oxidation (0.47 ± 0.04 mM H_2_S h^−1^) relative to WT PS (Fig. 1A), indicating that H_2_S oxidation does occur in the absence of a functional nitrate reductase. In contrast, a similar experiment performed with a nitrate-grown strain containing a deletion of the prerequisite PcrC perchlorate reductase subunit (Δ*pcrC*) (16) was incapable of H_2_S oxidation (Fig. 1B), further demonstrating the perchlorate-dependent nature of sulfide oxidation.

10.1128/mBio.02023-16.1 FIG S1  H_2_S oxidation with concomitant perchlorate reduction. Data represent PS cultures showing H_2_S oxidation (top panel) coupled to perchlorate reduction (bottom panel) in the absence of other electron donors. Download FIG S1, PDF file, 0.1 MB. Copyright © 2017 Mehta-Kolte et al. 2017 Mehta-Kolte et al. This content is distributed under the terms of the Creative Commons Attribution 4.0 International license.

### PSOX is a function of H_2_S concentration and cell density.

To understand the physiology of PSOX, we investigated the effects of H_2_S concentration and cell density independently on the rate and extent of the metabolism. A 10% inoculum from a late-log-phase culture of PS pregrown with lactate and perchlorate was transferred into minimal medium containing 10 mM perchlorate and H_2_S in a range of concentrations (2 to 10 mM) (Fig. S2A). As expected, PS completely oxidized sulfide at 2 and 4 mM over the course of 6 h. In contrast, although there was an excess of perchlorate, only partial H_2_S oxidation (4.14 mM ± 0.03) was observed at 8 mM and no oxidation occurred at 10 mM (Fig. S2A), suggesting a toxicity threshold for sulfide. Similar experiments using medium amended with 10 mM perchlorate and 2 mM H_2_S designed to determine the impact of cell density (Fig. S2B) revealed a specific H_2_S oxidation rate of ~1.09 × 10^−9^ mM cell^−1^ h^−1^. As expected, higher cell densities led to proportionally higher oxidation rates. However, a minimum cell density was essential to overcome the initial toxicity in order to achieve complete H_2_S oxidation by PS. At a low cell density (optical density at 600 nm [OD_600_] of 0.1), the cells were unable to oxidize 2 mM H_2_S. These results demonstrate that the rate and extent of sulfide oxidation are functions of the ratio of the H_2_S concentration to cell density, indicating a degree of toxicity of the sulfide to the cell.

10.1128/mBio.02023-16.2 FIG S2  H_2_S oxidation is directly proportional to cell density. The rate of H_2_S oxidation is dependent on the concentration of H_2_S and cell number as depicted by optical density. Higher rates are observed for lower concentrations of H_2_S with high cell OD values. Download FIG S2, PDF file, 0.1 MB. Copyright © 2017 Mehta-Kolte et al. 2017 Mehta-Kolte et al. This content is distributed under the terms of the Creative Commons Attribution 4.0 International license.

### H_2_S is preferentially utilized over lactate.

**FIG 2:**
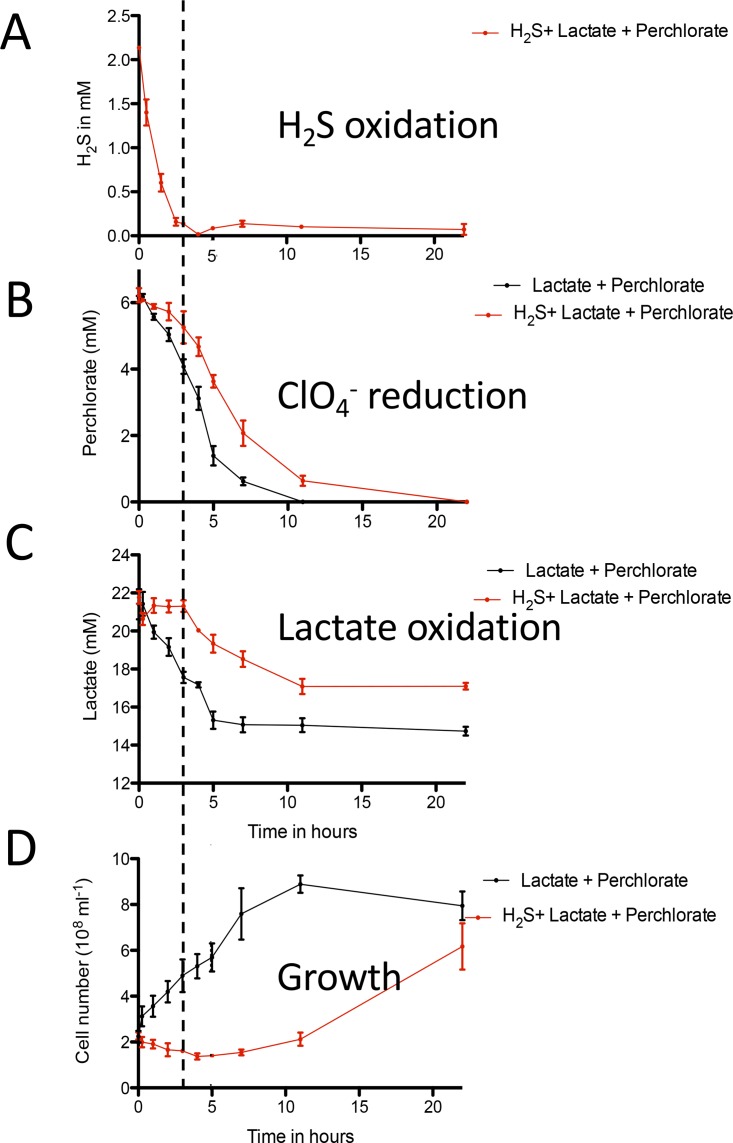
Kinetics of PSOX by DPRB. Data represent sulfide oxidation (A), perchlorate consumption (B), lactate consumption (C), and growth (D) in wild-type *Azospira suillum* culture amended with both lactate and sulfide as electron donors. The dashed line indicates when sulfide oxidation reached completion.

Previous studies (9) indicated that cell growth is not associated with H_2_S oxidation, even in the presence of 10 mM lactate, a preferred electron donor and carbon source for PS (9). Further investigation using cultures amended with both lactate and H_2_S revealed that no lactate consumption or cell growth occurred until H_2_S was completely oxidized at ~5 h (Fig. 2). Once H_2_S was depleted, lactate consumption with concomitant cell growth was observed (Fig. 2D), achieving a final cell density similar to that seen with the control cultures with no H_2_S amendment (~6.17 × 10^8^ cells ml^−1^; *P* = 0.06). The extended culture inhibition previously reported by Gregoire et al. (9) can be attributed to the pH fluctuations observed upon addition of sodium sulfide to poorly buffered growth medium (data not shown). This pH-dependent inhibition further explains the growth observed after the “stuck” culture was reinoculated into freshly buffered medium. All H_2_S oxidation experiments done in this study were performed using buffered minimal medium as described in Materials and Methods.

### H_2_S oxidation occurs downstream of the NADH dehydrogenase.

**FIG 3:**
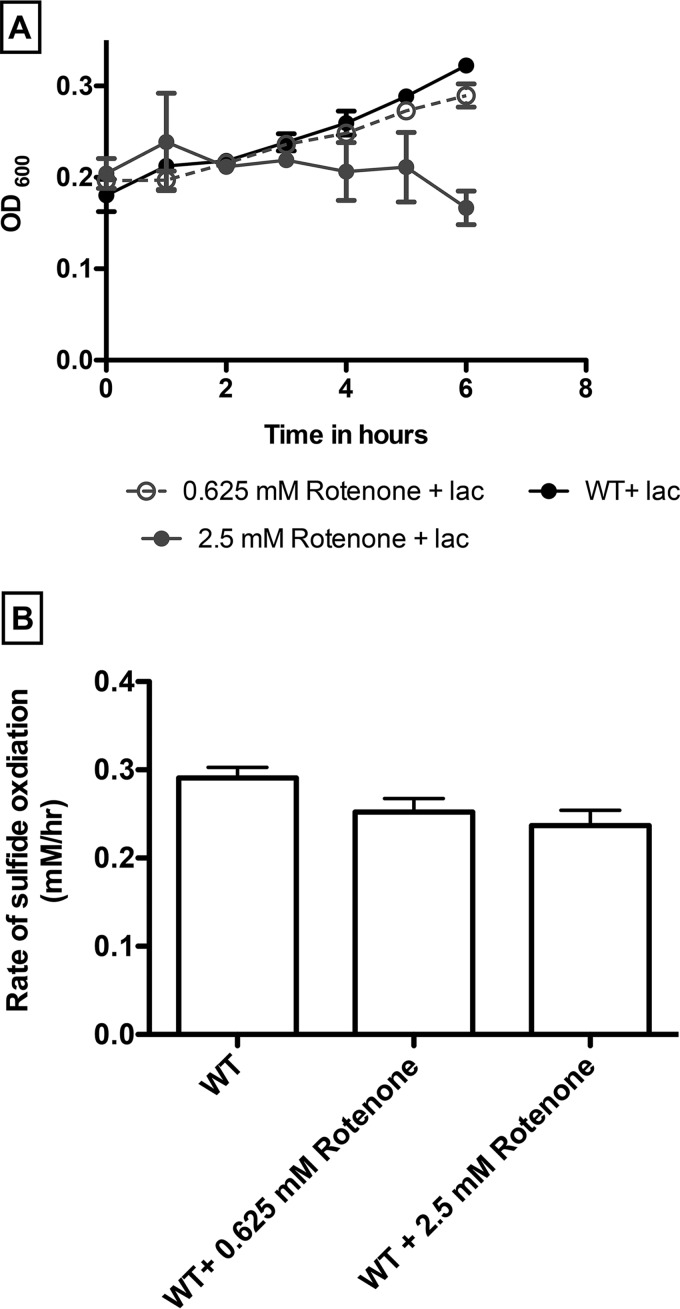
Growth (A) and sulfide oxidation (B) by wild-type *Azospira suillum* in the absence and presence of different concentrations of rotenone.

The perchlorate dependency of H_2_S oxidation suggests that the metabolism is directly associated with unique enzymes of the respiratory pathway or that a sulfide oxidase is selectively expressed in the presence of perchlorate. To determine which of these alternatives is the more likely, we investigated H_2_S oxidation with rotenone-treated cells. Rotenone is a specific inhibitor that blocks electron transfer between the NADH dehydrogenase (complex I) and coenzyme Q, preventing the utilization of NADH as a substrate at the start of the electron transport chain (ETC) (19–22). As expected, at a higher concentration (2.5 mM), rotenone effectively inhibited heterotrophic cell growth with lactate oxidation (Fig. 3A). In contrast, similar rotenone concentrations had no statistically significant impact (*P* = 0.01) on H_2_S oxidation (for 2.5 mM rotenone, 0.23 ± 0.01 mM H_2_S h^−1^; for 0.625 mM rotenone, 0.25 ± 0.01 mM H_2_S h^−1^) compared to untreated controls (0.29 ± 0.01 mM H_2_S h^−1^) (Fig. 3B). These results indicate that the presence of a functioning version of complex I is unnecessary for PSOX and suggest that H_2_S oxidation is mediated by a component downstream of complex I in the electron transport chain.

### “Omics” approach to identify the putative sulfide-oxidase.

To identify the genes responsible for H_2_S oxidation on a genome-wide scale, we performed saturated DNA bar-coded transposon mutant library sequencing on PS cells grown in the presence or absence of H_2_S. The barcode abundances were used to calculate the fitness values for individual genes (23, 24). Additionally, to obtain a comprehensive view of gene expression and protein synthesis patterns in response to H_2_S, transcriptomic analyses using transcriptome sequencing (RNA-seq), as well as differential proteomic profiling of PS cells, were performed and the results compared.

**FIG 4:**
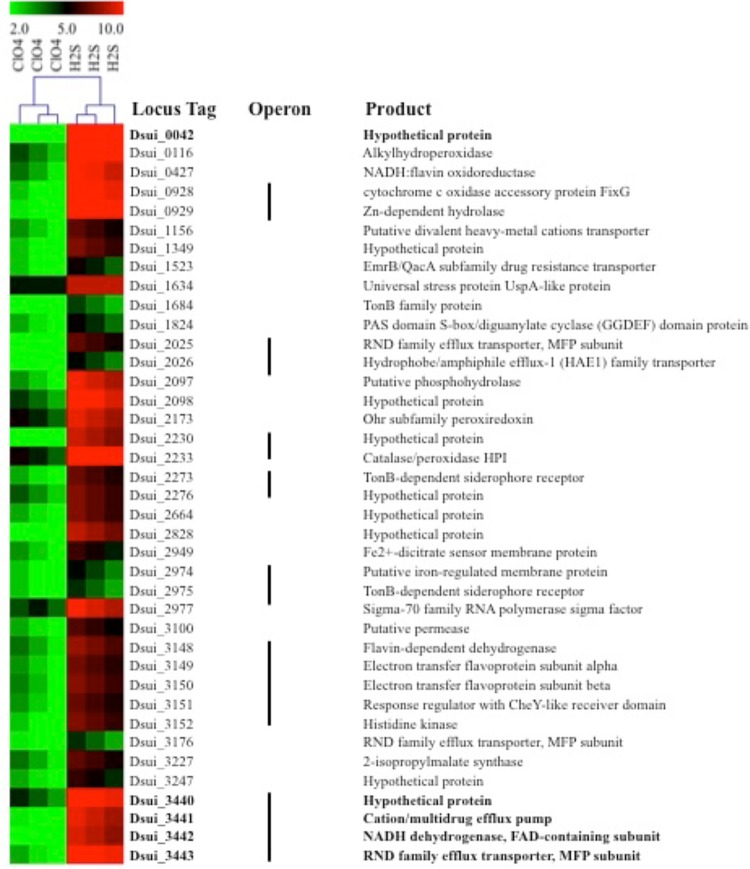
Heat plot of proteins differentially expressed under PSOX and heterotrophic culture conditions.

Random-barcode Tn-Seq (Bar-seq) fitness data suggested the involvement of a single operon (Dsui_3440-3443) (see Table S2 in the supplemental material), while comparative RNA-seq analysis revealed that this operon was highly expressed under H_2_S-oxidizing conditions (Fig. 4). This operon consists of genes encoding a protein of unknown function (Dsui_3440; *P* = 0.0003), a cation/multidrug efflux pump (Dsui_3441; *P* = 0.0002), a sulfide:quinone oxidase (SQR) (Dsui_3442; *P* = 0.0005), and an RND (Resistance-Nodulation-Division) family efflux transporter MFP subunit (Dsui_3443; *P* = 0.0001). This operon is particularly interesting, as the SQR (Dsui 3442) is a known alternative pathway for the H_2_S oxidation that is present in a diversity of prokaryotes (25). It is a 50-kDa subunit of a homodimeric membrane-associated protein harboring one non-covalently bound flavin adenine dinucleotide (FAD) cofactor (26). SQR belongs to the glutathione reductase family of flavoproteins, which have been shown to catalyze the two-electron oxidation of H_2_S to elemental sulfur and to reduce membrane-bound isoprenoid quinones (27, 28). This implies that SQR oxidizes and donates electrons from H_2_S to the electron transport chain at the level of the quinone pool, which is consistent with our observations from rotenone incubations (Fig. 3). Depending on the quinone species, the midpoint potential of the Q/QH_2_ couple is 100 to 300 mV more electropositive than the S^0^/H_2_S couple (26, 29). The resulting oxidized sulfur is released as a highly insoluble octameric ring (S_8_) or as short chains of polysulfide [HS-(S_n_)-SH], which are formed due to subsequent reaction between elemental sulfur and H_2_S. The sulfur is stored in either extracellular or intracellular protein-encapsulated elemental sulfur globules (30). Morphologically similar intra- and extracellular sulfur globules have previously been observed in H_2_S-oxidizing PS cultures (9), indicating that Dsui_3442 could be the putative H_2_S oxidase.

**FIG 5:**
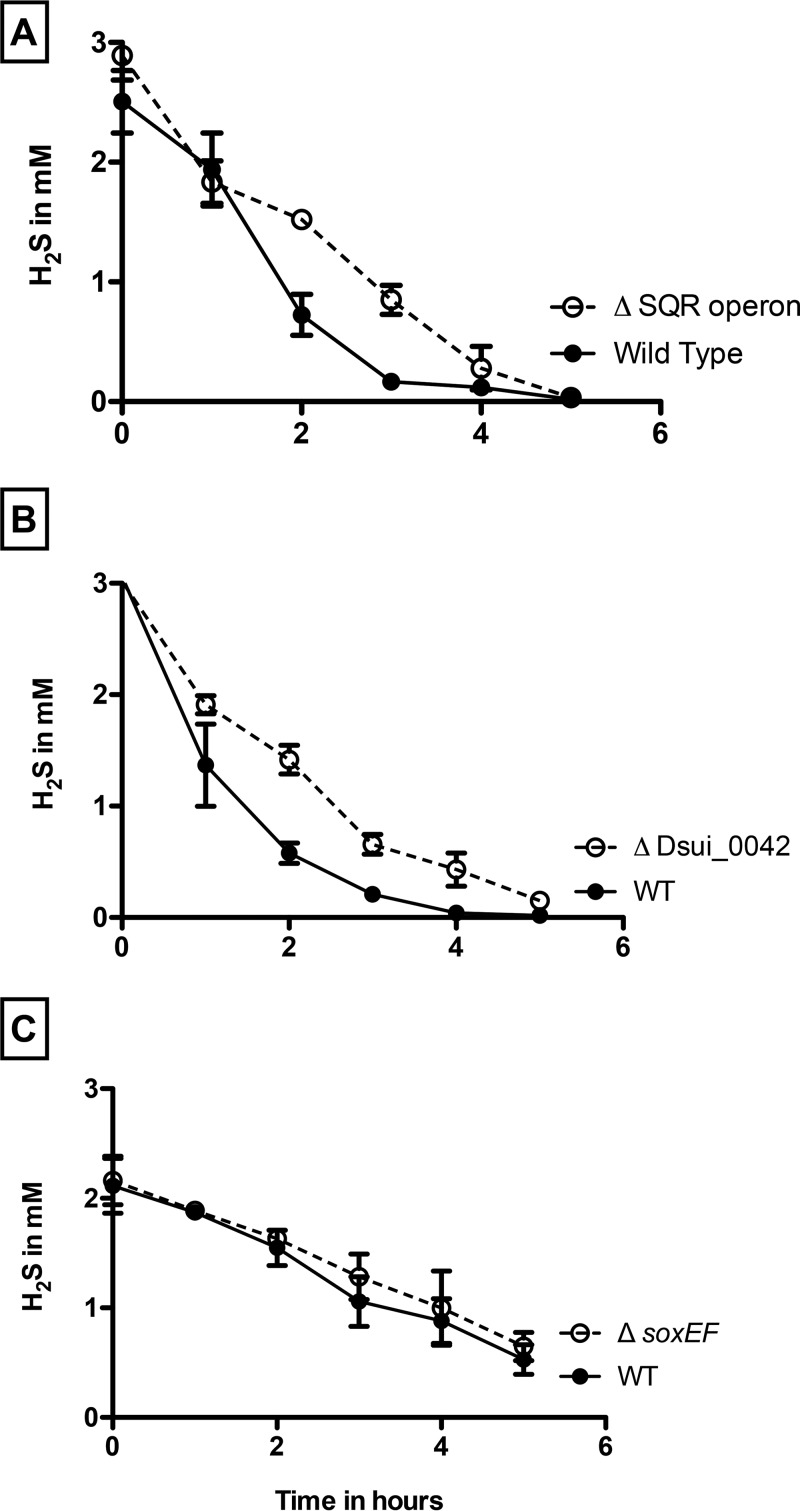
PSOX by washed cell suspensions of wild-type *Azospira suillum* in comparison to Δ*sqr* mutant (A), Δ*Dsui_0042* mutant (B), and Δ*soxEF* mutant (C).

**FIG 6:**
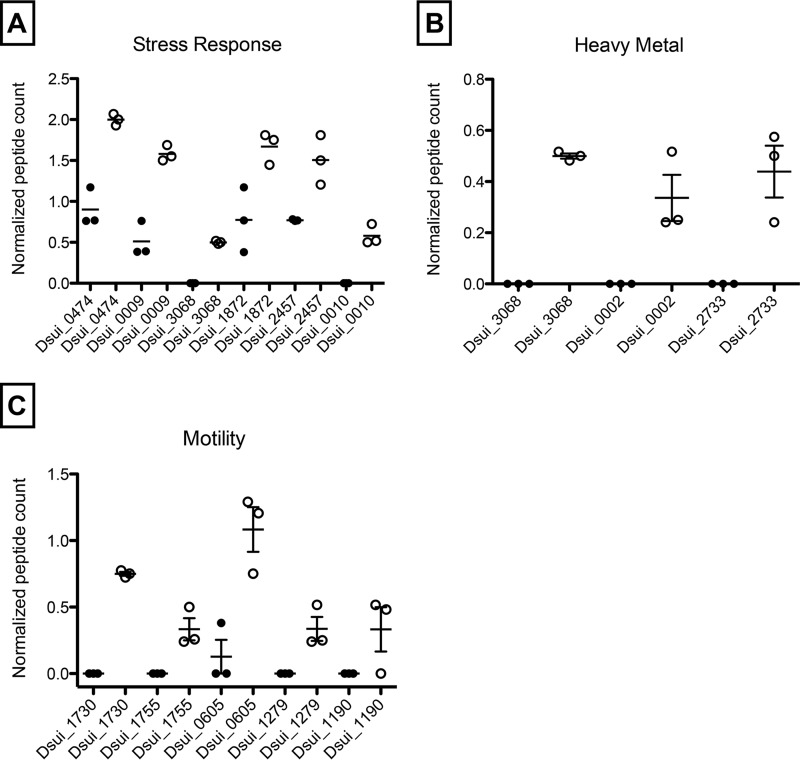
Differential expression of peptides associated with stress response (UspA—Dsui_0474, Dsui_1634; ClpA—Dsui_0009; RraA family protein—Dsui_0010; LepA—Dsui_2457) (A), heavy-metal efflux pumps (Fe^2+^-regulated membrane proteins/Fe uptake regulators—Dsui_0002, Dsui_2733, Dsui_3068) (B), and motility under PSOX and heterotrophic culture conditions (FlgM—Dsui_1730; flagellar motor switch protein FliG—Dsui_1755; PilT—Dsui_0605; ATPase PilB—Dsui_1279; PilP—Dsui_1190) (C).

To investigate the role of SQR in PSOX, a markerless in-frame deletion of Dsui_3442 was constructed as previously described (16). Phenotypic characterization revealed that the Δ*Dsui_3442* mutant showed no defect in the rate of H_2_S oxidation compared to the wild-type control (data not shown), suggesting that SQR is not involved in H_2_S oxidation. To determine whether other genes present in this operon could be important for H_2_S oxidation, an in-frame deletion mutant was constructed wherein the entire SQR operon (corresponding to Dsui_3440 to Dsui_3443) was deleted. The *ΔSQR* strain showed no significant difference in the rate of PSOX (0.49 ± 0.05 mM H_2_S h^−1^) compared to WT PS (0.50 ± 0.03 mM H_2_S h^−1^) (Fig. 5A). In addition to the RND family efflux transporter (Dsui_3443), RNA-seq and differential proteomic analysis identified other RND family efflux transporters (Dsui_1872, Dsui_2025, and Dsui_3176) that were also upregulated during PSOX (Fig. 4 and 6A and B). RND family transporters are widespread and catalyze the active efflux of many toxic compounds such as biocides and heavy metals (31, 32). Their upregulation in PSOX could be due to the chemical reactivity of H_2_S with different heavy-metal cations, potentially resulting in the toxic accumulation of metal sulfide complexes (32).

Other genes and proteins differentially expressed during PSOX included a universal stress protein (UspA) (Dsui_0474, Dsui_1634), an ATP-dependent Clp protease subunit (ClpA) (Dsui_0009), a RraA family protein (Dsui_0010), a GTP-binding elongation factor (LepA) (Dsui_2457), and various Fe^2+^-regulated membrane proteins/Fe uptake regulators (Dsui_0002, Dsui_2733, Dsui_2949, Dsui_2974, Dsui_3068) (Fig. 6). Taking the results in combination, upregulation of these genes and proteins suggests that exposure to H_2_S elicits a general stress response in PS, where proteins are denatured by either one or a combination of disruptions of disulfide bonds or reductions of metalloprotein redox centers. Exposure to H_2_S also often causes sequestering of Fe^2+^ (33–37), leading to upregulation of Fe regulatory proteins. Furthermore, proteomic analysis also indicated increased expression of various peptides involved in cell motility in PS, suggesting an evasion response to H_2_S.

One of the most abundant transcripts (Dsui_0042) in the RNA-seq experiment encoded a hypothetical protein (DUF2892) that was expressed at a level that was ~10-fold higher under PSOX conditions (*P* = 0.0009) (Table S3). As with other genes identified in the RNA-seq data set, incubation of an in-frame deletion mutant (the Δ*Dsui_0042* mutant) showed a minor impairment in the onset of H_2_S oxidation relative to the WT PS results; however, the rate of PSOX (0.55 ± 0.03 mM H_2_S h^−1^) was effectively identical to that observed for WT PS (0.55 ± 0.07 mM H_2_S h^−1^) (Fig. 5B). This suggests that Dsui_0042 may be involved in H_2_S-dependent stress responses or detoxification but that it is not a primary H_2_S oxidase. Taken together, the “omics” data identified genes and proteins involved in detoxification and a general stress response to H_2_S addition but failed to identify any prerequisite sulfide oxidoreductase.

### *soxE* and *soxF* are not required for HS^−^ oxidation coupled to perchlorate reduction in PS.

In addition to the differentially expressed SQR genes, PS has two additional known putative H_2_S oxidation genes. Dsui_1541 and Dsui_1540 encode SoxE and SoxF, respectively, which are predicted to be part of an operon. Both genes have previously been implicated in H_2_S oxidation in phylogenetically diverse chemolithotrophic sulfur-oxidizing microorganisms (2). SoxE is a monoheme *c*-type cytochrome (111 amino acids [aa]) and is the redox partner of SoxF. SoxF is a periplasmic monomer containing covalently bound flavin adenine dinucleotide (FAD), and homologues are ubiquitous in phototrophic and chemotrophic sulfur-oxidizing bacteria (38). SoxF homologues oxidize H_2_S to elemental sulfur or polysulfides (2).

To test the role of the *sox* genes, an in-frame double deletion of *soxE* and *soxF* (*ΔsoxEF*) was constructed. As previously observed with other mutants, the *ΔsoxEF* mutant readily oxidized sulfide (0.40 ± 0.02 mM H_2_S h^−1^) at rates similar to those seen with WT PS (0.42 ± 0.02 mM H_2_S h^−1^) (Fig. 5C). This result was not unexpected, as comparative genomics analyses of sequenced DPRMs revealed that *soxE* and *soxF* are not conserved regardless of the ability of the organisms to mediate PSOX (9). Furthermore, these genes were not implicated in H_2_S oxidation in our omics analyses (Fig. 4 and 6).

### H_2_S oxidation occurs directly at PcrAB.

During perchlorate respiration, the periplasmic perchlorate reductase (PcrAB) catalyzes two sequential 2e- transfer reductions (18) of perchlorate to chlorate (ClO_3_^−^) and of ClO_3_^−^ to chlorite (ClO_2_^−^). In the cell, the biogenic ClO_2_^−^ is dismutated by the periplasmic chlorite dismutase (Cld) into chloride and molecular oxygen and subsequently reduced to water by the cytoplasmic cytochrome *c* oxidase (10). In the absence of any apparent inducible H_2_S oxidase, and given that H_2_S oxidation is perchlorate dependent and was unaffected by complex I inhibition, we hypothesized that the perchlorate reductase could directly oxidize H_2_S.

**FIG 7:**
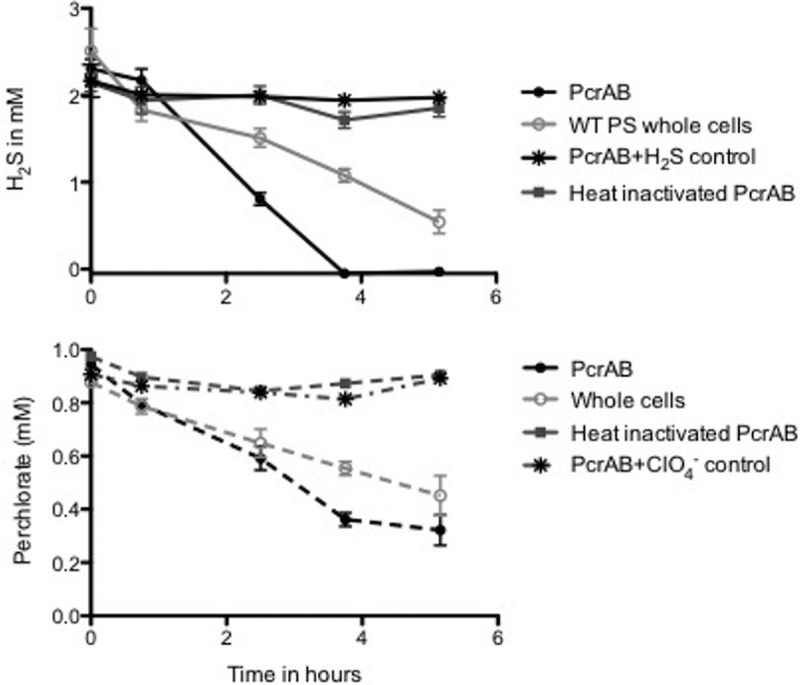
Sulfide oxidation (top panel) and perchlorate reduction (bottom panel) by wild-type *Azospira suillum* and purified perchlorate reductase enzyme.

To test this hypothesis, we purified PcrAB from WT PS cells and performed steady-state kinetic studies using H_2_S as the electron donor and perchlorate as the electron acceptor. Our results indicated that PcrAB independently mediated PSOX (0.52 ± 0.04 mM H_2_S h^−1^) (Fig. 7). No H_2_S oxidation occurred in control incubations lacking perchlorate or containing heat-inactivated PcrAB. Stoichiometric reduction of perchlorate consistent with the two-electron transfer previously reported for PSOX with whole cells (Fig. 7) (9) was observed. The steady-state PcrAB kinetics results for H_2_S oxidation were nearly identical with both perchlorate and chlorate as electron acceptors (Table 1). Recently, PcrAB has also been shown to alternatively turn over other oxyhaloanions (bromate, iodate, nitrate), and the *k*_cat_ value for nitrate (51.1 e^−^ min^−1^) was almost twice that for perchlorate (27.1 e^−^ min^−1^) (18). Consistent with this, and given the results of our whole-cell experiments outlined above, purified PcrAB also mediated H_2_S oxidation coupled to nitrate reduction (Table 1). However, the rate of nitrate-dependent H_2_S oxidation (NSOX) was approximately 5-fold lower than that observed with perchlorate (Table 1). Given the respective *k*_cat_ values, this was unexpected and was likely due in part to the reactive role of the intermediates formed during perchlorate respiration, (chlorite, molecular oxygen, and hypochlorite), each of which rapidly reacts abiotically with H_2_S (3). While nitrite produced as a result of PcrAB nitrate reduction can similarly react with H_2_S, the rate of the abiotic reaction between it and H_2_S is significantly lower than that observed with chlorite (Fig. S3).

10.1128/mBio.02023-16.3 FIG S3  Abiotic oxidation of H_2_S in the presence of chlorite or nitrite relative to unamended controls. Results demonstrate that H_2_S reacts much more rapidly with chlorite. Download FIG S3, PDF file, 0.1 MB. Copyright © 2017 Mehta-Kolte et al. 2017 Mehta-Kolte et al. This content is distributed under the terms of the Creative Commons Attribution 4.0 International license.

**TABLE 1:** Empirically determined kinetic parameters of purified PcrAB with H_2_S as the electron donor

Electron acceptor	*V*_max_ (nmol HS^−^/min)	*K*_*m*_ (mM)
Perchlorate	55.06 ± 19.48	2.99 ± 1.59
Chlorate	59.73 ± 10.02	2.06 ± 0.707
Nitrate	11.17 ± 7.18	0.584 ± 0.12

### Stable isotope analysis supports the concept of a hybrid enzymatic-abiotic metabolism.

**FIG 8:**
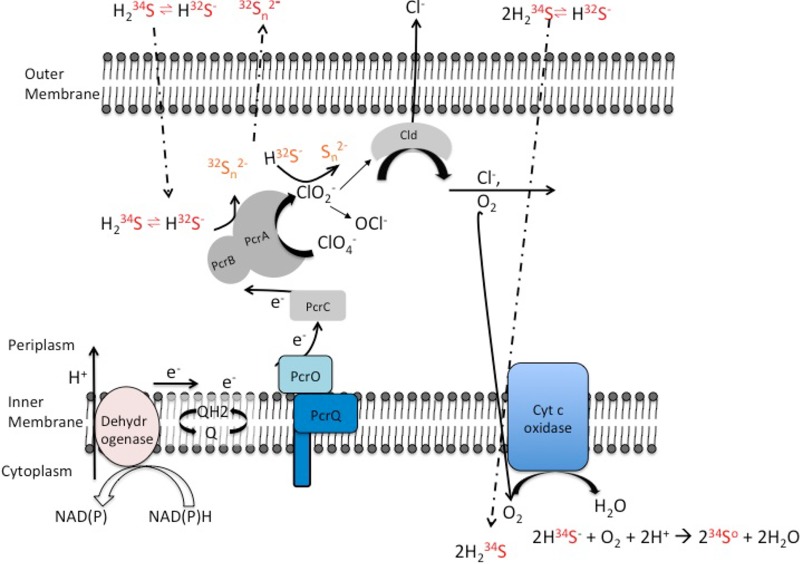
Model for H_2_S oxidation coupled to perchlorate reduction in wild-type *Azospira suillum*. PcrA, PcrB, PcrC, PcrO, and PcrQ are the functional subunits of the perchlorate reductase transferring electrons from the quinone pool (Q/QH_2_) to perchlorate. Cld, chlorite dismutase; Cyt c oxidase, cytochrome *c* oxidase; Dehydrogenase, NAD(P)H dehydrogenase component of complex I.

Stable isotope analysis of the ^34^S/^32^S ratio in both H_2_S and elemental sulfur species indicated that PS preferentially consumes the heavier isotope, accumulating the lighter isotope in the residual sulfide over the time course (Fig. S4). Rayleigh fractionation curve analysis of the data revealed an inverse isotopic fractionation factor of +1.8‰, which is consistent with that previously reported for this metabolism (9). The preferential uptake of the heavier ^34^S isotope is thought to be primarily due to the equilibrium partitioning between H_2_S and HS^−^ species in solution (39), resulting in H_2_S being enriched in ^34^S by 2.4 to 6.0‰ compared to HS^−^ (40, 41) and 1.2 to 3.0‰ compared to total dissolved sulfide at circumneutral pH (approximately equimolar concentrations of H_2_S and HS^−^ species). Preferential use of the H_2_S species over the HS^−^ species would therefore result in inverse isotopic fractionation. A possible mechanism to account for preferential H_2_S utilization is for the lipid-soluble isotopically heavier H_2_S species to pass through the cytoplasmic membrane and be oxidized to elemental sulfur by biogenic oxygen formed as an intermediate of perchlorate reduction, whereas the isotopically lighter polar HS^−^ is excluded and remains in the periplasm (Fig. 8). Periplasmic dissolved sulfide can be oxidized by PcrAB and/or pass back through the periplasmic membrane to rejoin the external sulfide pool, leading to lower δ^34^S levels in the dissolved sulfide pool external to the cell. This is analogous to the isotopic fractionation network suggested for microbial sulfate reduction described by Brunner et al. (42).

10.1128/mBio.02023-16.4 FIG S4  Isotopic fraction analysis of H_2_S oxidation by PS. Stable isotope analysis profiles of δ^34^S for HS^−^ and S0 during H_2_S oxidation by PS are shown. The rate of H_2_S oxidation coupled with the rate of perchlorate reduction is also shown for each of the data points for stable isotope analysis. Download FIG S4, PDF file, 0.1 MB. Copyright © 2017 Mehta-Kolte et al. 2017 Mehta-Kolte et al. This content is distributed under the terms of the Creative Commons Attribution 4.0 International license.

## DISCUSSION

The results of our studies demonstrate that sulfide oxidation by PS is specific to perchlorate respiration and occurs independently of complex I and upstream metabolic processes. Furthermore, the Bar-seq, RNA-seq, differential proteomics, and genetic studies indicated that a specific sulfide oxidoreductase is not involved in this metabolism. Rather, sulfide oxidation was identified as a result of short-circuiting of the electron transport chain, with H_2_S donating electrons directly to the PcrAB in the periplasm. The presented data are consistent with a model (Fig. 8) where H_2_S is oxidized by a hybrid enzymatic-abiotic mechanism similar to that proposed previously for nitrate-dependent Fe(II) oxidation (15) and represents a combination of two mechanisms operating under perchlorate-reducing conditions. First, H_2_S oxidation is enzymatically initiated through direct interaction with PcrAB coupled to the reduction of perchlorate to chlorite. Cld subsequently catalyzes the conversion of chlorite into chloride and molecular oxygen. Second, either or both the biogenic chlorite and oxygen further abiotically react with H_2_S, producing polysulfide, elemental sulfur, and Cl^−^. The amount of H_2_S oxidized by PS resulted in complete stoichiometric reduction of perchlorate to chloride. This is still consistent with the two-electron transfer and oxidation of H_2_S to polysulfide and elemental sulfur as previously observed (8, 9).

In our study, insignificant H_2_S oxidation coupled to nitrate reduction was observed when cells were pregrown with nitrate. However, perchlorate pregrown cells could oxidize H_2_S partially using nitrate as an electron acceptor, further validating that the perchlorate respiratory components (Pcr genes) are essential. Since these genes are predominantly expressed under perchlorate-reducing conditions (unpublished data), this pregrowth is required. Youngblut et al. recently showed that purified PcrAB can use nitrate at slightly higher rates than perchlorate (18), but H_2_S was not tested as an electron donor. In our work, only partial H_2_S oxidation was observed in both whole-cell studies (~1 mM) and enzymatic studies (11.17 ± 7.18 nmol H_2_S-min^−1^) under nitrate-reducing conditions. Furthermore, we demonstrated that nitrite does not abiotically oxidize H_2_S as rapidly as chlorite. As transient intermediates of perchlorate respiration, including chlorite, hypochlorite, and oxygen, have all been shown to rapidly react with H_2_S (43, 44), the observed PSOX rate represents a combination of the enzymatic rate and the abiotic rate of H_2_S oxidation. In contrast, the reaction between nitrite and H_2_S is much more recalcitrant, suggesting that the measured NSOX rate is primarily a function of the enzymatic H_2_S oxidation rate over the time course of the experiment. As purified PcrAB turns over nitrate and perchlorate at rates with similar orders of magnitude (18), this would suggest that the measured PSOX rate, which is 5-fold higher than the NSOX rate, is dominated by the abiotic reaction with the RCS intermediates.

No growth is associated with PSOX, which can be explained by the fact that H_2_S interacts directly with the PcrAB, keeping it in the reduced state and hindering electron transfer from the upstream electron transport chain (ETC) (Fig. 8). This was further evidenced by the inability of a complex I inhibitor to affect H_2_S oxidation and would imply that the rate of PSOX kinetics is higher than that of the upstream ETC. Taken together, these results imply a short-circuiting of the ETC and no proton motive force (PMF) generation during PSOX. In addition, the diffusion of H_2_S through the cytoplasmic membrane to abiotically react with biogenic O_2_ would result in a passive transfer of protons across the gradient with a resultant dissipation of any existing PMF (Fig. 8). Further evidence of this kinetic short-circuiting is seen in the fact that lactate oxidation resumed once H_2_S was depleted and that only then was growth observed.

A thorough investigation of H_2_S oxidation in PS using omics approaches determined that PSOX represents an inadvertent form of detoxification metabolism, as most of the genes or proteins expressed under conditions of H_2_S additions, such as the universal stress protein UspA and the RraA family protein, are associated with cellular stress. Also, in both RNA-seq and proteomic experiments, heavy-metal efflux pumps were highly represented, suggesting that these proteins are involved in the export of metal sulfides. This was further validated by upregulation of Fe^2+^ uptake proteins and bacterioferritin, implying that the cells were undergoing iron starvation in response to reduced intracellular free iron levels due to the iron-sequestering activity of H_2_S. None of the known sulfide oxidoreductase genes targeted in this study were shown to be important to PSOX.

### Conclusion.

In this study, we characterized the hybrid enzymatic-abiotic mechanism underlying PSOX mediated by the perchlorate reductase and the reactive intermediates generated innately during perchlorate respiration. Data demonstrating the ability of all tested DPRMs to oxidize H_2_S to elemental sulfur with concomitant reduction of perchlorate have been previously published (8, 9), and the results of the current study are consistent with this being an innate ability of all DPRMs. Although this process is thermodynamically favorable, no growth is observed, at least in the model betaproteobacterial DPRM *Azospira suillum* PS. This suggests that PSOX in PS is primarily an inadvertent detoxification mechanism carried out by PcrAB in addition to nonenzymatic reactions between H_2_S and the reactive intermediates (chlorite, oxygen) of the pathway.

## MATERIALS AND METHODS

### Bacterial strains, plasmids, and culture conditions.

*Azospira suillum* PS (ATCC BAA-33/DMS 13638) was revived from laboratory freezer stocks and used as the wild-type strain for all genetic manipulations as well as H_2_S oxidation experiments. PS was routinely grown at 37°C either in aerobic ALP medium (16) or in anoxic minimal medium with 20 mM sodium lactate or 2 mM Na_2_S (unless stated otherwise) as the electron donor and 10 mM perchlorate (NaClO_4_) or 10 mM nitrate (NaNO_3_) as the electron acceptor. One liter of anoxic minimal medium contains 0.98 g NaH_2_PO_4 ⋅ _2H_2_O, 1.94 g Na_3_PO_4_, 0.1 g of KCl, 0.25 g NH_4_Cl, and 10 ml of both vitamin mix and mineral mix as previously described (16). This medium is adjusted with higher concentrations of phosphate salts to account for the pH fluctuations seen with the previous medium recipe upon H_2_S addition (9), as discussed in Results. All media were adjusted to pH 7.2, made anoxic by being flushed with oxygen-free N_2_, and sealed with butyl rubber stoppers prior to autoclaving. Strains used for cloning and conjugation purposes are listed in Table S1 in the supplemental material. The plasmids constructed and the primers used in this study are described in Table S1. All genetic manipulations were performed as described by Melnyk et al. (16).

10.1128/mBio.02023-16.5 TABLE S1  Bacterial strain, primers, and plasmids used in this study. This table describes all the strains used in and constructed for this study. Download TABLE S1, XLSX file, 0.01 MB. Copyright © 2017 Mehta-Kolte et al. 2017 Mehta-Kolte et al. This content is distributed under the terms of the Creative Commons Attribution 4.0 International license.

10.1128/mBio.02023-16.6 TABLE S2  Replicate-averaged gene fitness values and standard error for barcode analysis by sequencing (Bar-seq) experiment. Data represent results of Bar-seq analysis of PS cultures grown with 1 mM H_2_S or mid-log-phase cultures spiked with 1 mM H_2_S compared to no-stressor controls. Download TABLE S2, XLSX file, 1.3 MB. Copyright © 2017 Mehta-Kolte et al. 2017 Mehta-Kolte et al. This content is distributed under the terms of the Creative Commons Attribution 4.0 International license.

10.1128/mBio.02023-16.7 TABLE S3  (Tab A) All proteomics data sorted by accession number. (Tab B) Proteomics data sorted by *P* value for 2-fold values or higher. Data represent PS proteins displaying the greatest increase in peptide counts in H_2_S-added cultures compared to no-H_2_S-added control cultures. Total numbers of peptides observed in all samples for a given peptide, ratios of normalized peptides observed in both cultures, and *P* values from Student’s *t* test are reported. Download TABLE S3, XLSX file, 0.1 MB. Copyright © 2017 Mehta-Kolte et al. 2017 Mehta-Kolte et al. This content is distributed under the terms of the Creative Commons Attribution 4.0 International license.

10.1128/mBio.02023-16.8 TABLE S4  Differentially expressed genes from the RNA-seq experiments. Download TABLE S4, XLSX file, 0.02 MB. Copyright © 2017 Mehta-Kolte et al. 2017 Mehta-Kolte et al. This content is distributed under the terms of the Creative Commons Attribution 4.0 International license.

### H_2_S oxidation experiments.

A 10% (vol/vol) inoculum of approximately 3 × 10^8^ cells ml^−1^ was used in all growth experiments, which were performed in anaerobic tubes using pure cultures of PS at 37°C. In all H_2_S experiments, 2 mM sodium sulfide was used as an electron donor to study H_2_S oxidation, except in the experiment where the effect of H_2_S concentrations (1 mM to 10 mM) on H_2_S oxidation was studied. Cell suspension assays were performed using late-log-phase cells grown on anoxic minimal medium with lactate and perchlorate, and the suspensions were centrifuged for 10 min, washed three times in basal medium, and resuspended in basal medium in anaerobic serum bottles. Cells were then injected into triplicate tubes at the start of the experiment using a degassed syringe. H_2_S concentrations were quantified using a modification of the Cline assay (45) and were read at 660 nm on a Varian Cary 50 Bio spectrophotometer equipped with a Cary 50 MPR microplate reader (Varian). Sulfur isotope analysis of H_2_S was performed on sacrificial time point triplicates in screw top 4-ml wash vials (Agilent Technologies part number 5182-0551) to minimize the headspace and hence the volatilization of H_2_S.

Growth in cultures was monitored by direct counts of acridine orange-stained cells. At various time points, 500 μl of cultures was fixed in 3.7% formaldehyde. Cells were then diluted in water, and 1 ml of the diluted cells was stained with acridine orange at a final concentration of 1 μg/ml. A glass vacuum filter apparatus (Fisher) was used to apply the samples onto polycarbonate black filters (Maine Manufacturing; part no. 1215609). The filters were then mounted on microscope slides using ProLong antifade diamond mountant to suppress photobleaching and to preserve the signal of stained cells. Cells were counted by microscopic inspection with an oil immersion lens at ×100 magnification using an Axioimager M1 microscope (Zeiss).

For rotenone-mediated NADH dehydrogenase inhibition, PS cells were grown as described above with addition of either 0.625 mM or 2.5 mM from an ethanol stock solution of rotenone. Controls were incubated with ethanol only. H_2_S oxidation assays were performed after preincubation of cells with rotenone for 2 h.

### Analytical techniques.

Perchlorate was measured by ion chromatography on an ICS-1500 system using an AS9-HC anion-exchange column (Dionex; Thermo Electron North America, Sunnyvale, CA) with a 35 mM sodium hydroxide mobile phase at a flow rate of 1 ml min^−1^. Lactate concentrations were measured by high-performance liquid chromatography (HPLC) (Dionex model LC20), using a UV-visible light detector (Dionex AD20) at 210 nm and an Aminex HPX-87H (Bio-Rad) column with a mobile phase of 0.008 M H_2_SO_4_ and a flow rate of 0.9 ml min^−1^.

Fractionation of sulfur isotopes was investigated by analyzing the ratios of ^34^S to ^32^S present in residual H_2_S. A 0.2-ml volume of 0.5 M silver nitrate was used to precipitate out the dissolved sulfides. The silver sulfide (Ag_2_S) formed was purified with 1 ml of 3% ammonium hydroxide and washed with deionized water. All samples were dried at 60°C overnight before being loaded together with V_2_O_5_ for isotope analysis. Sulfur isotope ratios were measured using a Costech ECS4010 elemental analyzer in helium continuous-flow mode interfaced with a Thermo Delta V Plus isotope ratio mass spectrometer. Isotope ratios are reported in standard delta (δ^34^S) notation (units of per million [‰]). The 1σ reproducibility for δ^34^S was ±0.2‰.

### RNA-seq—growth conditions, sample preparation, and data analysis.

Minimal medium was used with 20 mM lactate and 10 mM perchlorate for RNA-seq experiments. Replicate anaerobic cultures (six) were initiated by inoculating fresh anoxic minimal medium with aerobic cells to a starting OD at 600 nm of 0.08. These were grown for approximately three doublings, at which point 2 mM sodium sulfide was spiked into three cultures to initiate sulfide oxidation for 4 h (complete oxidation of H_2_S is observed between at 4 and 5 h). Cells were subsequently collected for RNA extractions from all cultures. Cells were centrifuged and resuspended in Trizol (Life Technologies, Inc.) and extracted according to the manufacturer’s instructions. Extracted RNA was treated twice with DNase I (Thermo Scientific) to remove any DNA contamination and purified using an RNeasy minikit. rRNA removal (Ribo-Zero rRNA removal kit; Epicentre), cDNA synthesis, library preparation (WaferGen PrepX RNA kit), and 100-bp single-end sequencing (HiSeq2000; Illumina) were performed at the Vincent J. Coates Genomic Sequencing Laboratory (Berkeley, CA). The raw sequence data generated were filtered to discard any reads that did not meet overall quality values as determined by the FastQC tool in the Galaxy server. The sequences were used as an input in FASTQ format for subsequent analysis, and gene expression levels were quantified in samples incubated in the presence and absence of H_2_S.

RNA-seq analysis of sequence data was performed using the Arraystar QSeq application of the DNAStar Lasergene Genomic Suite (DNASTAR, Inc., Madison, WI, USA). Input files for the application were the FASTQ sequence read files and a GenBank file containing a reference genome for PS. FASTQ reads were mapped against the reference genome using the QSeq program to quantify gene expression levels in the absence or presence of H_2_S. Mapped read count normalization was applied to the data based on the number of reads per kilobase of coding sequence per million mapped reads (RPKM) (46). Expression levels were considered significant only when the log_2_ RPKM value was ≥8. The normalized RNA-seq data were used to construct heat maps generated using MeV (47).

### Proteomics—growth conditions, sample preparation, and data analysis.

Experiments were performed in replicate cultures using the same media and experimental growth conditions as described for the RNA-seq experiments, except the starting inoculum (OD at 600 nm) was 0.04 and 50-ml volumes of mid-log-phase cultures with and without H_2_S additions were used. Cells were centrifuged and pelleted anoxically at 4,000 × *g*, the supernatant was decanted, and the pellet was resuspended in 100 mM ammonium bicarbonate at pH 7.4. Cells were sonicated and digested with Trypsin Gold (Promega) prior to liquid chromatography-tandem mass spectrometry (LC-MS/MS) analysis as described previously (48). For proteomic data analysis, normalized peptide counts were used as a semiquantitative measurement of relative protein abundances. Peptide counts were normalized by dividing the number of peptides for a given gene by the total number of peptides present for that sample. Student’s *t* test was used to compare the normalized peptide counts for the two conditions, and *P* values of <0.05 were considered to be significant (15).

### Random-barcode Tn-Seq.

Frozen aliquots (1 ml) of PS tagged-transposon pools stored at −80°C (23) were recovered in oxic 50 ml minimal medium containing kanamycin as a selection marker at an initial OD at 600 nm of 0.02. The initial time zero (T0) cells were harvested at an OD at 600 nm of ~0.6 (~5 doublings) and transferred into anoxic minimal medium containing lactate (20 mM) and perchlorate (10 mM) with and without H_2_S (2 mM) at an initial OD of 0.03. Growth was monitored by measuring OD at 600 nm using a Spectronic 20D spectrophotometer. When pools reached an OD at 600 nm of ~0.8 (~5 doublings), 1-ml aliquots were collected by centrifugation and stored at −20°C for genomic DNA extraction. For the spike experiment, 2 mM H_2_S was added when pools reached an OD at 600 nm of 0.6 and the pools were harvested after 5 h.

Genomic DNA was extracted with a DNeasy kit (Qiagen) following the protocol for extraction of genomic DNA from Gram-negative bacteria. The optional RNase treatment step was included. DNA barcodes were then PCR amplified and sequenced as previously described (23).

Strain fitness was calculated as previously described (49) as the log_2_ ratio of the abundance after growth versus the abundance at the start of the experiment. Gene fitness data represent averages of the strain fitness values. Gene fitness values were further normalized by subtracting gene fitness values for no-stress controls from gene fitness values for stress experiments. Thus, reported fitness values in Table S2 are log_2_ values (stress data/no-stress control data). Genes with fitness values greater than 1 were considered beneficial mutations, and those with fitness values below −1 were considered detrimental mutations.

### PcrAB expression, purification, and sulfide oxidation analysis.

Active PcrAB from wild-type PS cells was purified and concentrated anoxically to 7 mg ⋅ ml^−1^ using a three-column purification protocol as recently described by Youngblut et al. (18). Steady-state kinetic analysis was done for H_2_S oxidation experiments with 0.2 μM of purified PcrAB using 300-µl Reacti-vials (Thermo Scientific) in an anaerobic chamber (Coy Labs). PcrAB was inactivated by incubation at 80°C for 10 min. H_2_S concentrations were measured using the modified Cline assay (45). Perchlorate concentrations were measured as described in the “Analytical techniques” section.

### Data availability affirmation.

We confirm that the data represented in the manuscript accurately reflect the raw data collected and will ensure that the original data are preserved and retrievable for at least 6 years following publication.
