# Supplementary material for: Mechanism of H2S Oxidation by the Dissimilatory Perchlorate-Reducing Microorganism Azospira suillum PS
Source: mBio. 2017 Feb 21;8(1):e02023-16. doi: 10.1128/mBio.02023-16 (PMC5358917; doi:10.1128/mBio.02023-16)
Supplement: FIG S1 [file mbo001173198sf1.pdf]

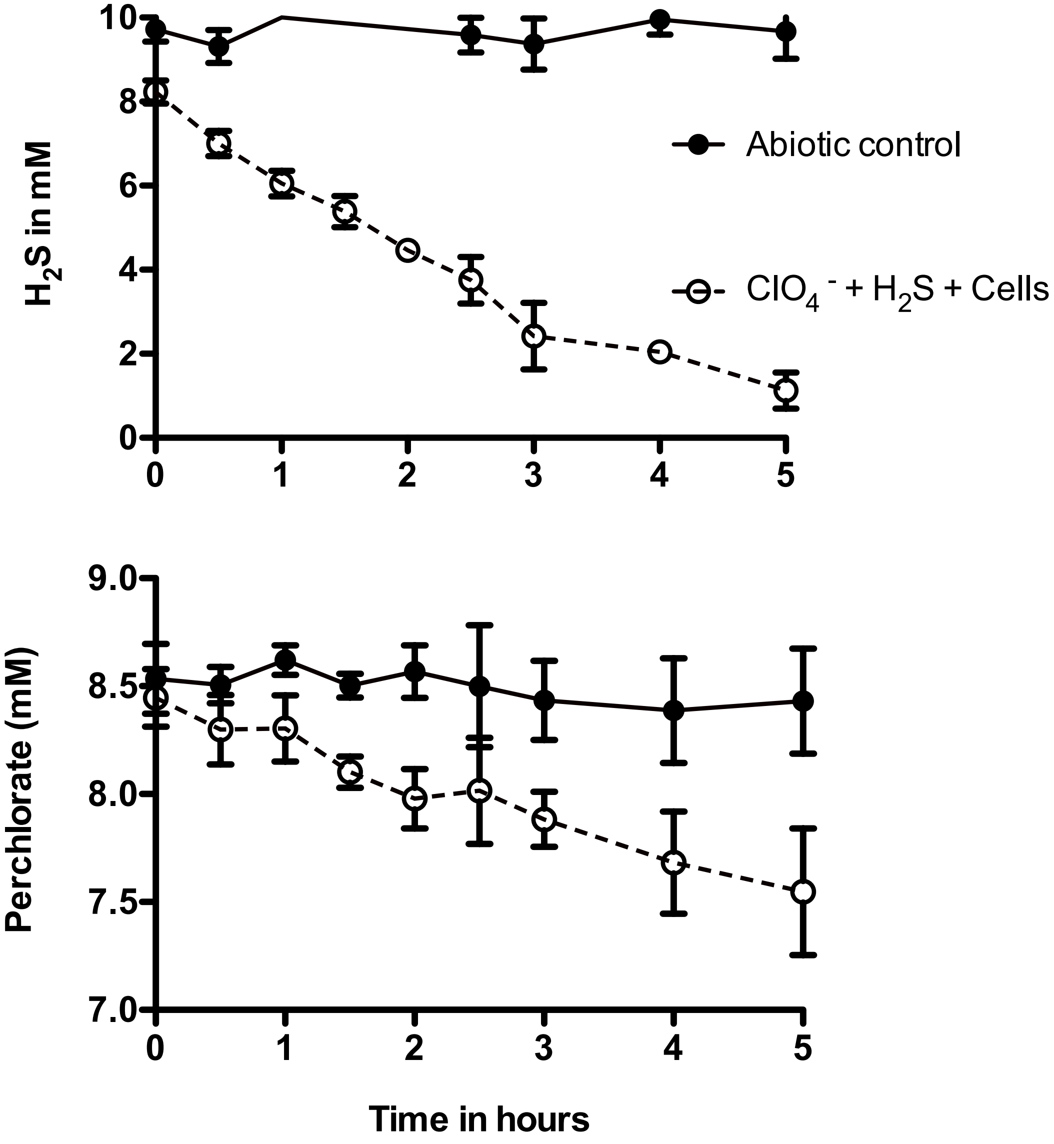

Figure S1. H_2S oxidation with concomitant perchlorate reduction. PS cultures showing H_2S oxidation (top panel) is coupled to perchlorate reduction (bottom panel) in absence of other electron donors.
