# Supplementary material for: Mechanism of H2S Oxidation by the Dissimilatory Perchlorate-Reducing Microorganism Azospira suillum PS
Source: mBio. 2017 Feb 21;8(1):e02023-16. doi: 10.1128/mBio.02023-16 (PMC5358917; doi:10.1128/mBio.02023-16)
Supplement: FIG S2 [file mbo001173198sf2.pdf]

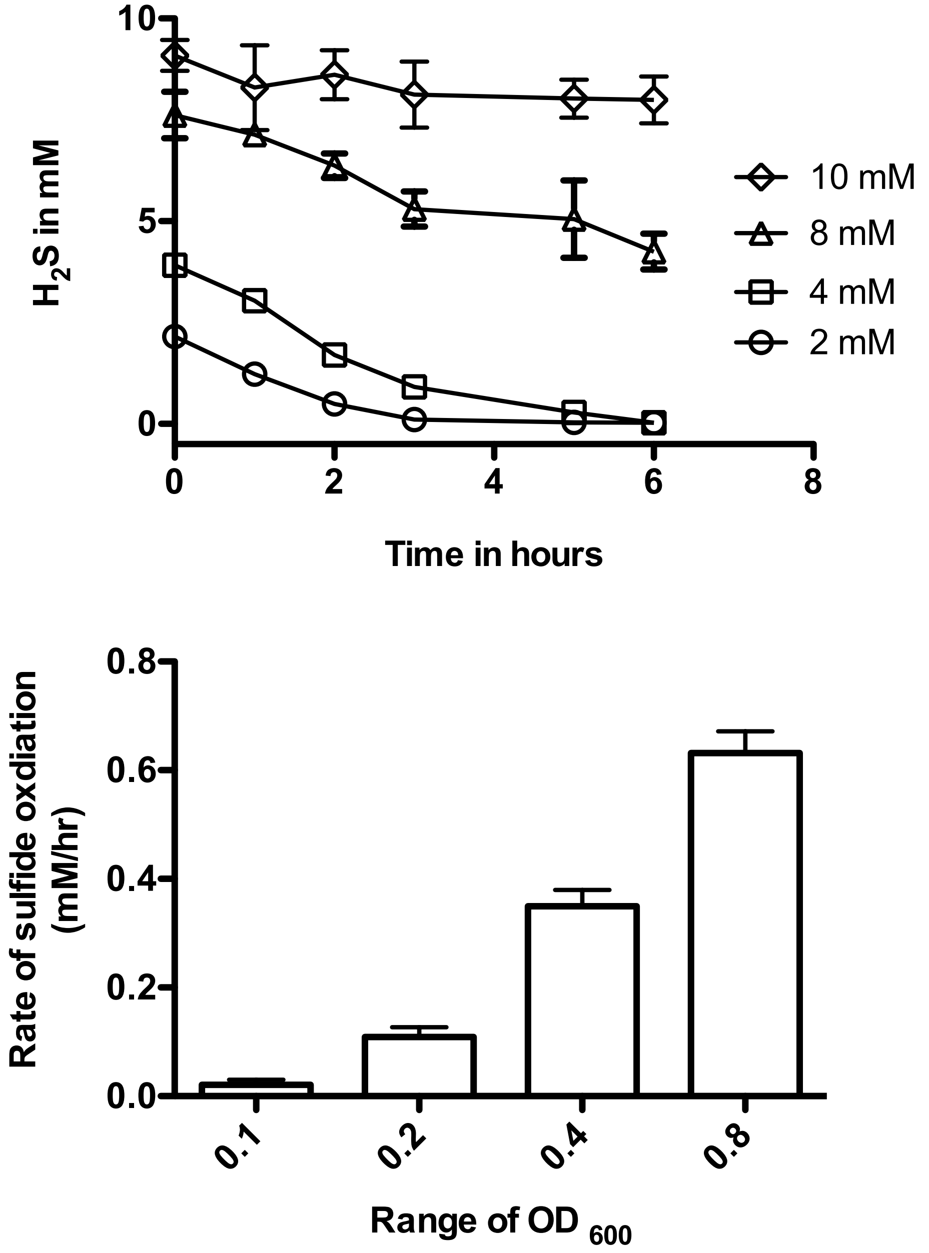


**Figure S2 $H_2S$ oxidation is directly proportional to cell density**. The rate of $H_2S$ oxidation is dependent on the concentration of $H_2S$ and cell OD, faster rates are observed for lower concentrations of $H_2S$ with high cell OD.
