# Supplementary figures and images for: Mechanism of H2S Oxidation by the Dissimilatory Perchlorate-Reducing Microorganism Azospira suillum PS
Source: mBio. 2017 Feb 21;8(1):e02023-16. doi: 10.1128/mBio.02023-16 (PMC5358917; doi:10.1128/mBio.02023-16)

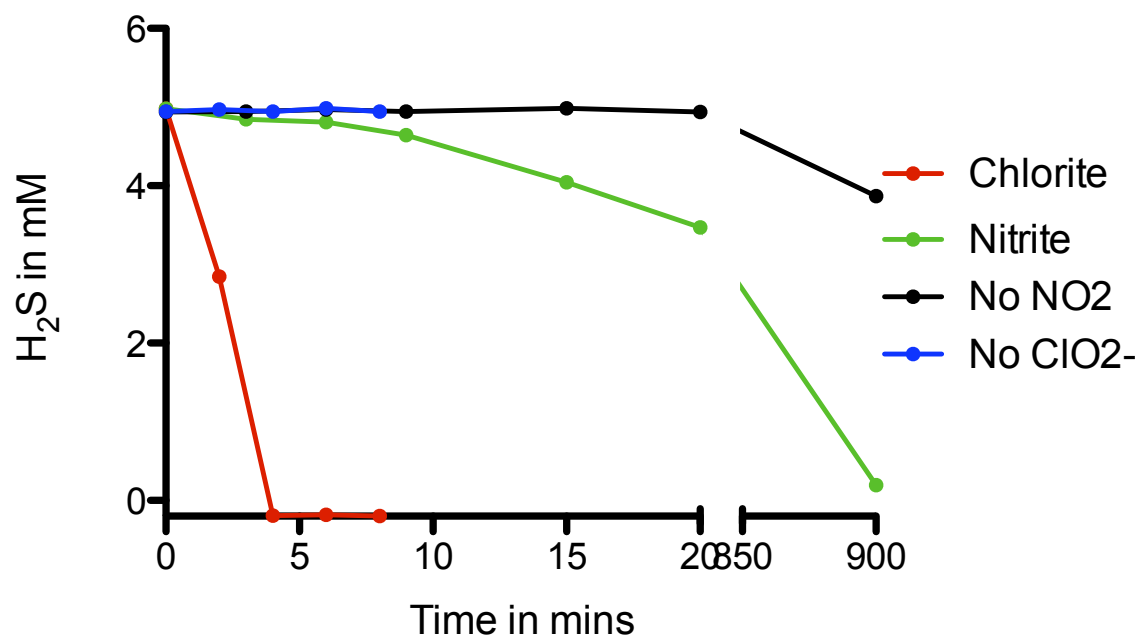

**SI Figure 3.** Abiotic H<sub>2</sub>S oxidation with chlorite and nitrite in comparison to controls.

Supplement: FIG S3 [file mbo001173198sf3.pdf]
