# Supplementary material for: Mechanism of H2S Oxidation by the Dissimilatory Perchlorate-Reducing Microorganism Azospira suillum PS
Source: mBio. 2017 Feb 21;8(1):e02023-16. doi: 10.1128/mBio.02023-16 (PMC5358917; doi:10.1128/mBio.02023-16)
Supplement: FIG S4 [file mbo001173198sf4.pdf]

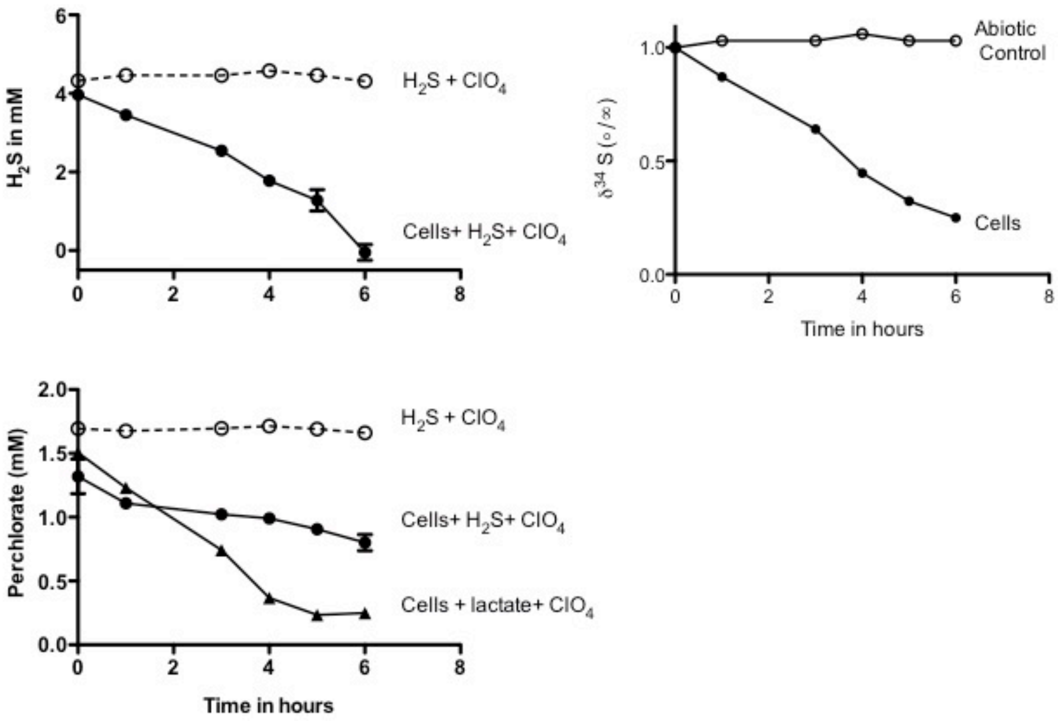

Figure S4 Isotopic fraction analysis of H_2S oxidation by PS. Stable isotope analysis profiles of $\delta^{34}S$ for HS^- and S^0 during H_2S oxidation by PS. The rate of H_2S oxidation with the rate of perchlorate reduction is also shown for each of the data points for stable isotope analysis.
